# Surge in macrolide dispensing to Swiss children in a period of increased incidence of *Mycoplasma pneumoniae* detection: an interrupted time-series analysis

**DOI:** 10.1093/jacamr/dlaf123

**Published:** 2025-07-11

**Authors:** Manon Jaboyedoff, Catherine Plüss-Suard, Patrick M Meyer Sauteur, Stephen P Jenkinson, Alessandro Cassini, Noémie Boillat-Blanco, Pierre Alex Crisinel, François Angoulvant

**Affiliations:** Unit of Pediatric Infectious Diseases and Vaccinology, Service of Pediatrics, Department Women-Mother-Child, Lausanne University Hospital and University of Lausanne, Lausanne, Switzerland; Swiss Centre for Antibiotic Resistance, Institute for Infectious Diseases, University of Bern, Bern, Switzerland; Division of Infectious Diseases and Hospital Epidemiology, Children’s Research Center, University Children’s Hospital Zurich, University of Zurich, Zurich, Switzerland; Institute of Primary Health Care (BIHAM), University of Bern, Bern 3012, Switzerland; Swiss Pharmacists Association PharmaSuisse, Bern-Liebefeld 3097, Switzerland; Cantonal Doctor Office, Public Health Department, Canton of Vaud, Lausanne, Switzerland; Service of Infectious Diseases, Department of Internal Medicine, Lausanne University Hospital and University of Lausanne, Lausanne, Switzerland; Service of Infectious Diseases, Department of Internal Medicine, Lausanne University Hospital and University of Lausanne, Lausanne, Switzerland; Unit of Pediatric Infectious Diseases and Vaccinology, Service of Pediatrics, Department Women-Mother-Child, Lausanne University Hospital and University of Lausanne, Lausanne, Switzerland; Service of Pediatrics, Department Women-Mother-Child, Lausanne University Hospital and University of Lausanne, Lausanne, Switzerland

## Abstract

**Objectives:**

To evaluate the trend in macrolide ambulatory use among children in Switzerland following a global surge in *Mycoplasma pneumoniae* infections in late 2023.

**Methods:**

We conducted a population-based interrupted time-series analysis of macrolide use in Swiss children aged 0 to 11 years from 2018 to 2023 using national ambulatory antibiotic claims data. The main outcome was the evolution of macrolide use in ambulatory setting, expressed as monthly defined daily doses (DDD) per 1000 children. We defined two time periods: (i) the pre-autumn 2023 period, before *M. pneumoniae* detections increased in Switzerland (1 January 2018 to 30 September 2023) and (ii) the autumn 2023 period, after *M. pneumoniae* detections increased in Switzerland (1 October 2023 to 31 December 2023). We built a quasi-Poisson regression model to estimate the changes in macrolide monthly DDD per 1000 children from October 2023 in Switzerland. The model accounted for temporal trends before the WHO alert and for the seasonal pattern of macrolide prescriptions. We analysed amoxicillin use as a control outcome.

**Results:**

We found a significant increase in macrolides use expressed in monthly DDD per 1000 children aged 0 to 11 years from October 2023 in Switzerland [+235% (95%CI +139%–+368%), *P* value < 0.001]. The use of amoxicillin remained stable in both age groups after October 2023.

**Conclusions:**

We found a 3-fold increase in macrolide monthly DDD per 1000 children in autumn 2023 in the context of a global increase in *M. pneumoniae* infections. Monitoring macrolide resistance and promoting appropriate prescription practices are essential.

## Introduction

Macrolides are the first-line treatment for infections caused by *Mycoplasma pneumoniae* in children. *M. pneumoniae* infections occur worldwide but were quasi-absent during and following the COVID-19 pandemic.^1,2^ A marked increase in *M. pneumoniae* infections occurred globally at the end of the summer 2023, prompting the WHO to issue an alert in November 2023 informing about the upsurge of respiratory illnesses among children in Northern China.^3–5^ In children, *M. pneumoniae* causes respiratory tract infections, including community-acquired pneumonia (CAP), which generally manifests with milder symptoms and signs compared to other conventional bacterial causes of CAP. The main burden of disease caused by *M. pneumoniae* is therefore expected to be found in outpatient settings. We hypothesized that increased incidence of *M. pneumoniae* associated with the raised awareness of *M. pneumoniae* infections among paediatricians might have led to a higher rate of macrolide prescriptions.

We aimed to analyse macrolide paediatric claims data from Swiss community pharmacies invoices. The objective of the study was to evaluate trends in macrolide use in children in the context of *M. pneumoniae* cases increase in children by interrupted time-series analysis.

## Methods

### Study design

We conducted a population-based interrupted time-series analysis of macrolide antibiotic use in Swiss pharmacies over 72 months (1 January 2018 to 31 December 2023).

The main outcome was the evolution of macrolide (clarithromycin and azithromycin) use in ambulatory setting, expressed as monthly defined daily doses (DDD) per 1000 children. The interruption was set on 1 October 2023, when *M. pneumoniae* detections increased in Switzerland, coinciding with the first media alerts about the upsurge of respiratory illnesses among children.^3,6^ We analysed amoxicillin as a control outcome to prevent potential confounding. Given that amoxicillin is not effective to treat *M. pneumoniae* infections due to its natural resistance against β-lactam antibiotics, their increased incidence should not impact use of this antibiotic agent.

### Data collection and source

In Switzerland, antibiotics for children are dispensed only with a physician’s prescription. In the ambulatory setting, antibiotics can be delivered by community pharmacies and by self-dispensing physicians in some areas of the country. The Swiss Pharmacists Association (https://pharmasuisse.org) provides antibiotic (ATC J01) claims data, which are dispensed in community pharmacies, to ANRESIS, the Swiss Centre for Antibiotic Resistance (https://www.anresis.ch). Pharmacies participating in this antibiotic use surveillance system are those using the billing service of the professional association of Swiss pharmacists (ofac, https://www.ofac.ch), representing ∼50% of all pharmacies in Switzerland (ranging from 50% in 2023 to 53% in 2018). These pharmacies are distributed across the entire country and include both urban and non-urban areas, providing a geographically and demographically representative sample. Claims data are provided in DDDs aggregated by month and by age groups, i.e. for children: 0 to 1 years and 2 to 11 years of age.

DDD is a technical unit allocated to drugs by the WHO Collaborating Centre in Oslo in association with the WHO International Working Group on Drug Statistics Methodology. It represents the ‘assumed average maintenance dose per day for a drug used for its main indication in adults’. The main outcome unit, DDD, has not been developed for evaluation of drug consumption in children and does not allow comparison of drug use between age groups. However, DDD can be used for overall comparison including time-trends. We expressed DDD per 1000 children per month by using population estimates of the Swiss Federal Office of Statistics, adjusted by age group and by year.

### Statistical analysis

We built a quasi-Poisson regression model to estimate the changes in macrolide monthly DDD per 1000 children from October 2023 in Switzerland. We defined two time periods to analyse: (i) the pre-autumn 2023 period, before *M. pneumoniae* detections increased in Switzerland (1 January 2018 to 30 September 2023) and (ii) the autumn 2023 period, after *M. pneumoniae* detections increased in Switzerland (1 October 2023 to 31 December 2023). The model accounted for temporal trends before the WHO alert and for the seasonal pattern of macrolide prescriptions using harmonic terms (sines and cosines with 12-months periods). The time-unit was 1 month. We used data collected before October 2023 to generate counterfactual estimation of the number of DDD per 1000 children in the autumn 2023 period. We analysed data for the entire population of children and by age groups (i.e. 0 to 1 years and 2 to 11 years). We performed three sensitivity analyses to assess the robustness of the study findings: (i) clarithromycin use only, (ii) azithromycin use only and (iii) amoxicillin use as a control outcome. Analyses were performed using R Statistical Software (v.4.4.2; R Core Team 2021).

### Ethics

This study was based on aggregated data submitted to ANRESIS. Because of the anonymous nature of the data, neither ethical approval nor written informed consent from patients was required.

## Results

**Figure 1.**
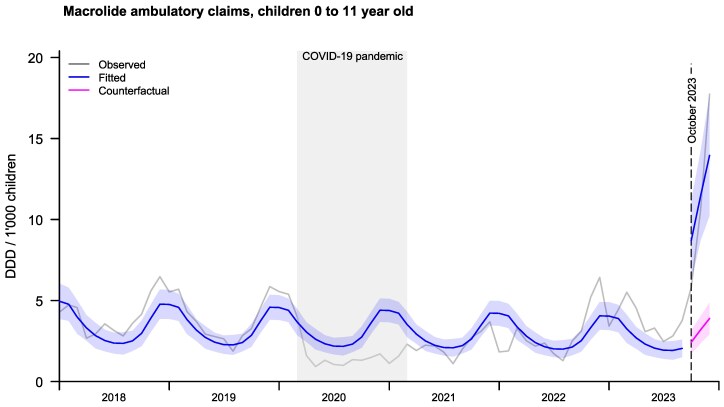
Monthly ambulatory DDDs of macrolides (clarithromycin and azithromycin) per 1000 children aged 0 to 11 years. The grey lines show the observed data. The blue line shows the fitted model based on observed data, with a blue shaded area showing the 95% CI. The pink line shows the expected DDD based on data before October 2023, with the pink shaded area showing the 95% CI.

We found a statistically significant increase in macrolides use expressed in monthly DDD per 1000 children aged 0 to 11 years from October 2023 in Switzerland [+235% (95%CI +139%–+368%), *P* value < 0.001], Figure 1. The increase was more pronounced in the age group 2 to 11 years [+246% (95%CI +146%–+386%), *P* value < 0.001] than in the age group 0 to 1 year old [+123% (95%CI +58%–+215%), *P* value < 0.001] [Supplementary material, Figures S1 and S2 (available as Supplementary data at *JAC-AMR* Online)].

The increase was higher for clarithromycin [+289% (95%CI +169%–+462%), *P* value < 0.001] than for azithromycin [+145% (95%CI +83%–+228%), *P* value < 0.001] when considering the entire population of children 0 to 11 years. In the age group 0 to 1 year old, there was an increase in clarithromycin use [+170% (95%CI +86%–+291%), *P* value < 0.001] but not in azithromycin use [+20% (95%CI −19%–+77%), *P* value 0.357) (Figures S3–S8).

The use of amoxicillin remained stable in both age groups after October 2023 (Figures S9–S11).

## Discussion

We found that macrolide antibiotic monthly DDD per 1000 children increased by ∼3-fold in Swiss children in autumn 2023. This surge followed the increase of cases of *M. pneumoniae* following the lifting of restrictions to reduce the impact of COVID-19 pandemic, which was observed globally and prospectively surveyed also for Switzerland by the European Society of Clinical Microbiology and Infectious Diseases Study Group for Mycoplasma and Chlamydia Infections (ESGMAC) MAPS study.^2,3^ Other factors than treatment of *M. pneumoniae* pneumonia may have contributed to the increase in macrolide consumption. First, *M. pneumoniae* carriage may be common in children, and awareness of the increased incidence of *M. pneumoniae* infections among paediatricians could have led to increased diagnostic testing.^7^ Consequently, this could have resulted in the treatment of *M. pneumoniae* infections beyond pneumonia. Second, there are other indications for prescribing macrolides to children such as infections with *Bordetella pertussis* or *Chlamydia pneumoniae*, cat scratch disease, bacterial diarrhoea or beta-lactam allergies. The increased incidence of pertussis in Europe in 2023/24 and *C. pneumoniae* compared to the pandemic years could therefore also partly explain the rise in macrolide consumption, especially in the younger age group.^8,9^ In addition, the transient increase in macrolide claims during winter 2022 could partly be related to the group A *Streptococcus* surge reported during that period. Third, amoxicillin shortage observed globally could have led to increased macrolide use in replacement.^10^ This is, however, unlikely in our population as amoxicillin use remained stable.

The increase in azithromycin consumption was less pronounced compared to the increase in clarithromycin consumption. This trend may reflect adherence to expert recommendations favouring clarithromycin over azithromycin for the treatment of *M. pneumoniae* CAP, because of the notably long half-life of azithromycin.^11^ Clarithromycin and azithromycin are both in the ‘Watch’ group of ‘AWaRe’, the WHO classification of antibiotics, defined as broader-spectrum antibiotics with a higher potential of developing resistance. Macrolide consumption is an important driver for macrolide resistance and is associated with increased macrolide resistance in several pathogen including streptococci.^12^ It could also be associated with macrolide-resistant *M. pneumoniae* (MRMP).^13^ MRMP are highly prevalent in some countries, and infections are associated with longer illness, more severe disease and/or the presence of extrapulmonary manifestations than infections with macrolide-sensitive *M. pneumoniae*.^14,15^ Prudent use of macrolides is therefore essential to mitigate the potential risk of increasing antibiotic resistance and to ensure the continued effectiveness of these treatments.

### Limitations

Our analysis is based on claims data of ∼50% of community pharmacies in Switzerland and does not consider all antibiotic sales channels such as self-dispensing physicians. There was, however, no change over time in data collection methods. The short period of data available after *M. pneumoniae* re-emergence limits our ability to assess whether macrolide use returned to baseline levels. In addition, we did not have access to information on treatment indication, which limits the interpretation of age-specific trends.

### Conclusions

Macrolides DDD for children surged significantly in Switzerland in autumn 2023. The more pronounced increase in clarithromycin, despite the greater convenience of azithromycin, suggests that national guidelines may have supported appropriate antibiotic selection. The overall rise in macrolide use underscores the need for continuous monitoring of macrolide resistance and promotion of appropriate prescription practices.

## Supplementary Material

dlaf123_Supplementary_Data

## Data Availability

The datasets presented in this article are not publicly available. Requests to access the datasets should be directed to the corresponding author.

## Supplementary data

Figures S1 to S11 are available as Supplementary data at *JAC-AMR* Online.
