# Supplementary material for: Surge in macrolide dispensing to Swiss children in a period of increased incidence of Mycoplasma pneumoniae detection: an interrupted time-series analysis
Source: JAC Antimicrob Resist. 2025 Jul 11;7(4):dlaf123. doi: 10.1093/jacamr/dlaf123 (PMC12247048; doi:10.1093/jacamr/dlaf123)

Supplementary material

Surge in macrolide dispensing to Swiss children in a period of increased incidence of *Mycoplasma pneumoniae* detection: An interrupted time-series analysis

Table of content

[Macrolide ambulatory consumption by age group 2](#_Toc196987942)

[Figure S1 2](#_Toc196987943)

[Figure S2 2](#_Toc196987944)

[Clarithromycin ambulatory consumption by age group 3](#_Toc196987945)

[Figure S3 3](#_Toc196987946)

[Figure S4 4](#_Toc196987947)

[Figure S5 4](#_Toc196987948)

[Azithromycin ambulatory consumption by age group 5](#_Toc196987949)

[Figure S6 5](#_Toc196987950)

[Figure S7 6](#_Toc196987951)

[Figure S8 6](#_Toc196987952)

[Amoxicillin ambulatory consumption by age group (control outcome) 7](#_Toc196987953)

[Figure S9 7](#_Toc196987954)

[Figure S10 8](#_Toc196987955)

[Figure S11 8](#_Toc196987956)

# Macrolide ambulatory consumption by age group

Monthly ambulatory Defined Daily Doses (DDDs) of macrolide per 1’000 children aged 0 to 1 year (Figure A4) and 2 to 11 years (Figure A5).

The grey lines show the observed data. The blue line shows the fitted model based on observed data, with blue shaded area showing the 95% confidence intervals. The pink line shows the expected DDD based on data before October 2023, with pink shaded area showing the 95% confidence intervals.

## Figure S1

**
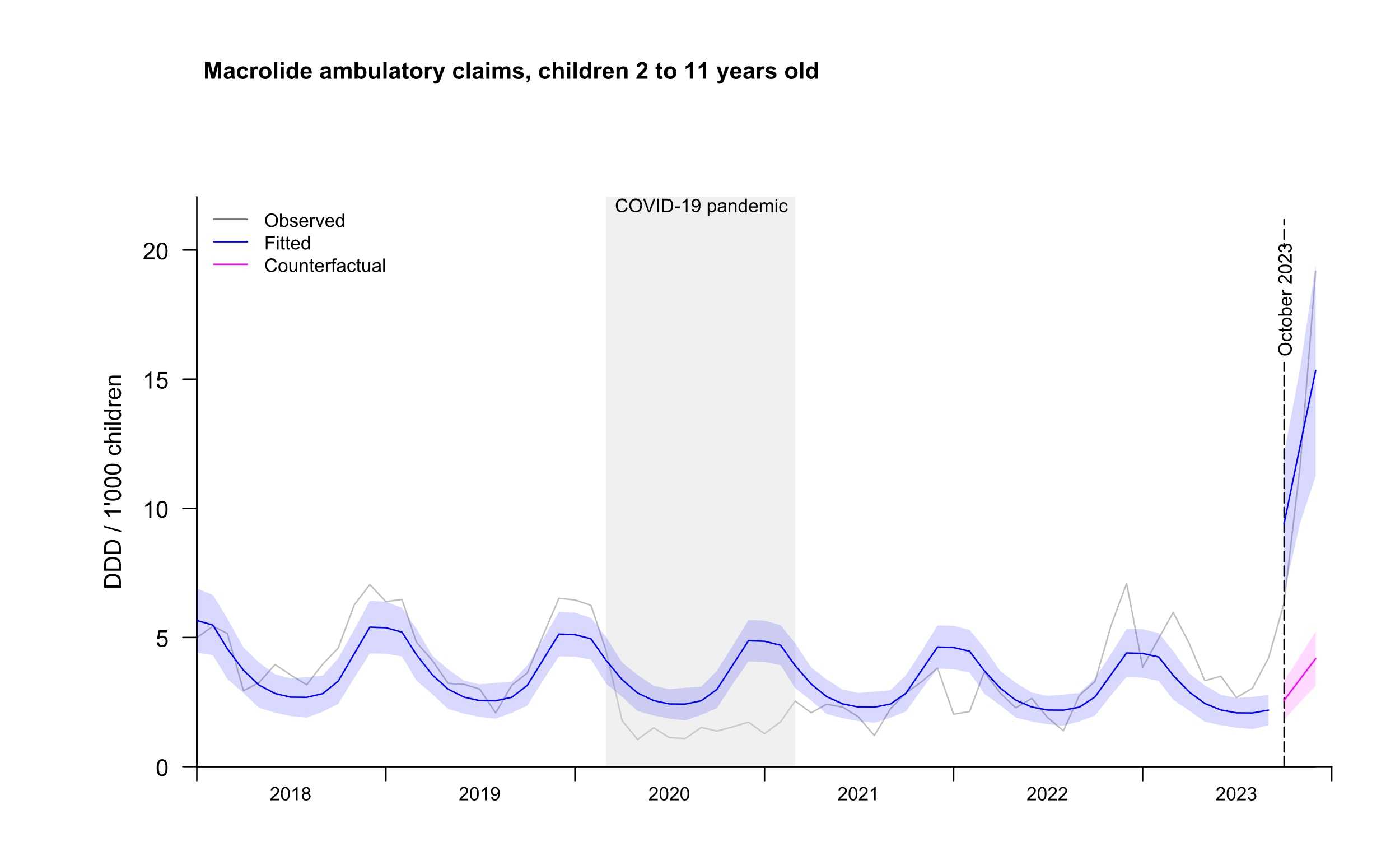
**

## Figure S2


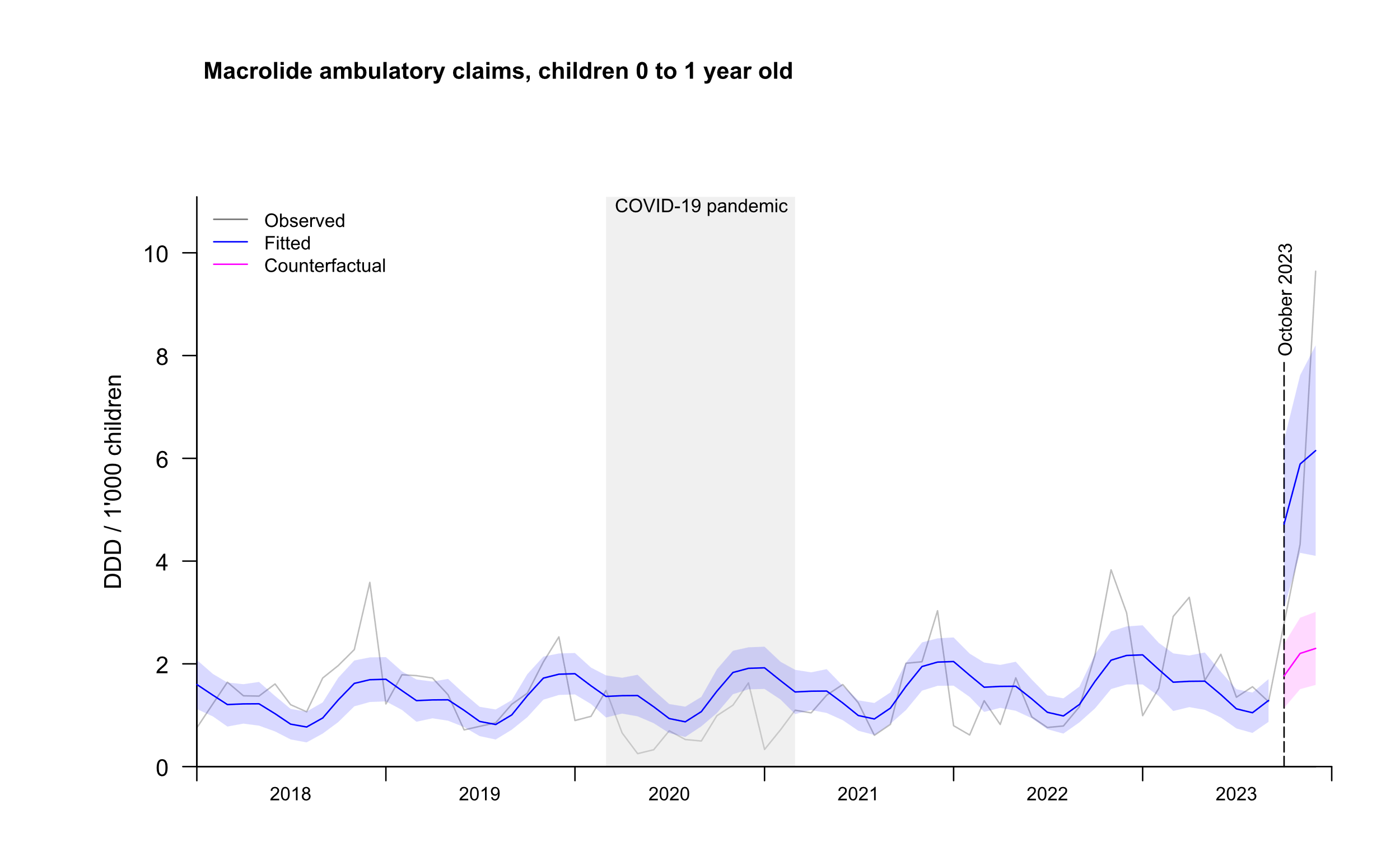


# Clarithromycin ambulatory consumption by age group

Monthly ambulatory Defined Daily Doses (DDDs) of clarithromycin per 1’000 children aged 0 to 11 years (Figure A5), 0 to 1 year (Figure A6) and 2 to 11 years (Figure A7).

The grey lines show the observed data. The blue line shows the fitted model based on observed data, with blue shaded area showing the 95% confidence intervals. The pink line shows the expected DDD based on data before October 2023, with pink shaded area showing the 95% confidence intervals.

## Figure S3


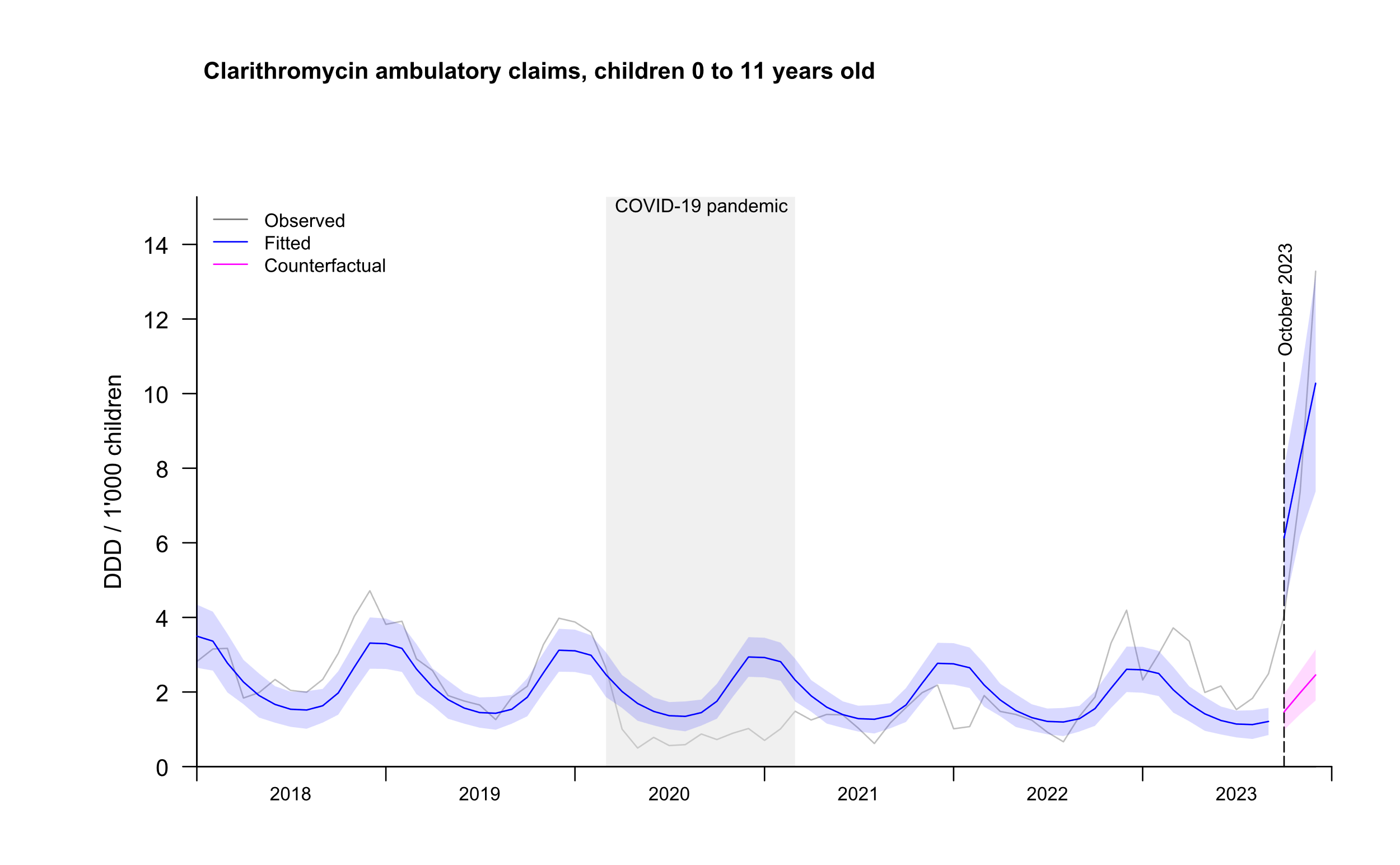


## Figure S4


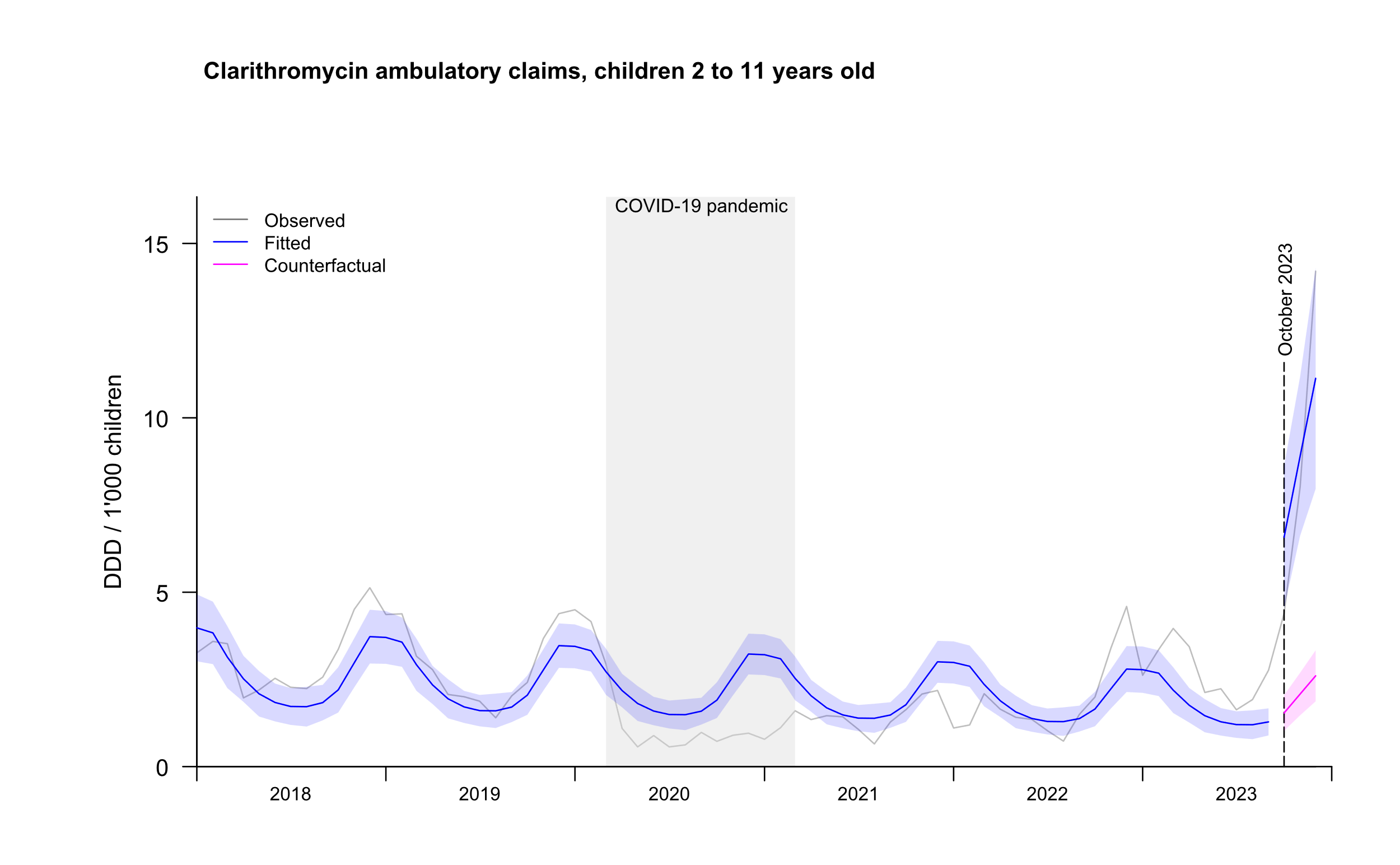


## Figure S5


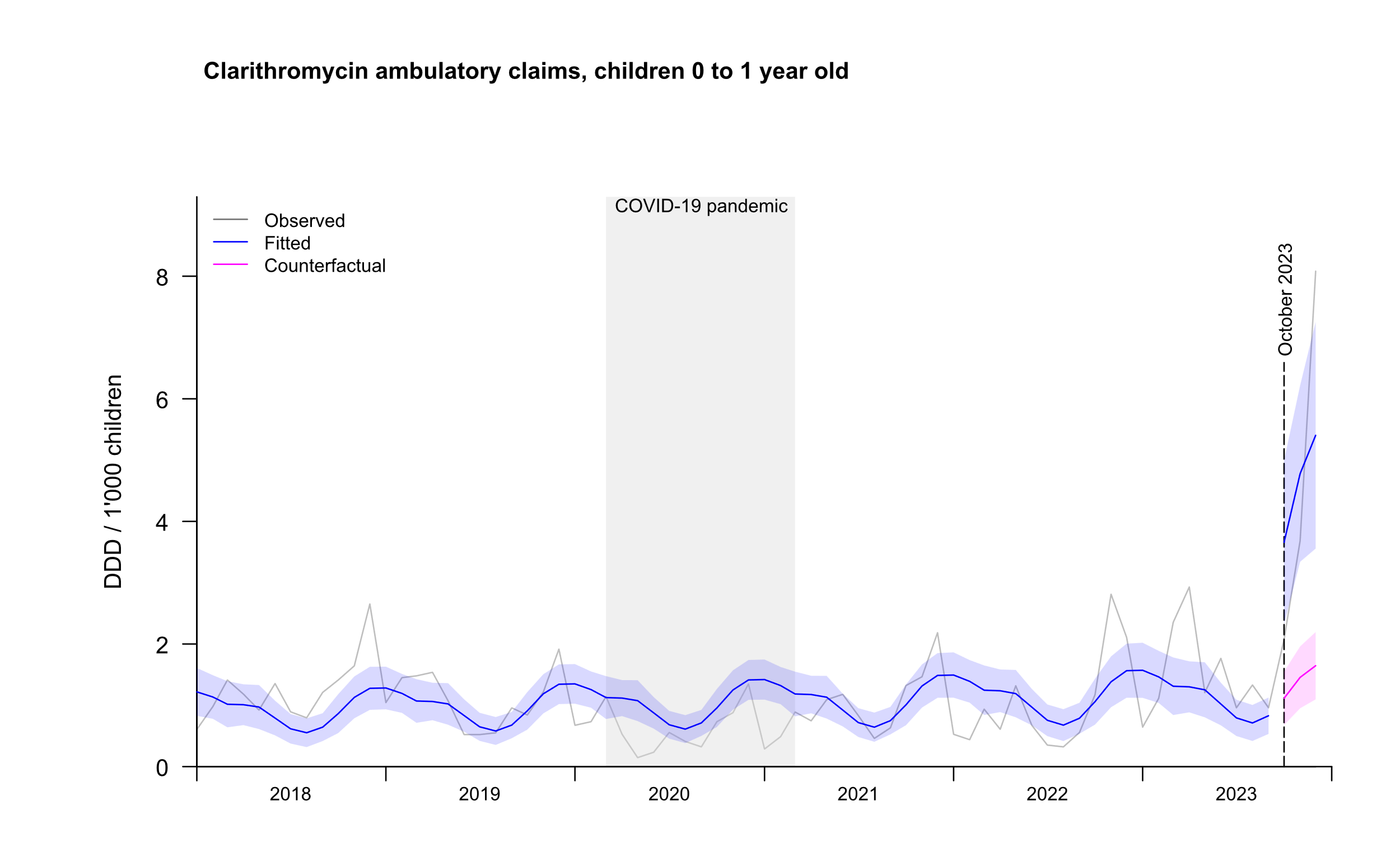


# Azithromycin ambulatory consumption by age group

Monthly ambulatory Defined Daily Doses (DDDs) of azithromycin per 1’000 children aged 0 to 11 years (Figure A8), 0 to 1 year (Figure A9) and 2 to 11 years (Figure A10).

The grey lines show the observed data. The blue line shows the fitted model based on observed data, with blue shaded area showing the 95% confidence intervals. The pink line shows the expected DDD based on data before October 2023, with pink shaded area showing the 95% confidence intervals.

## Figure S6


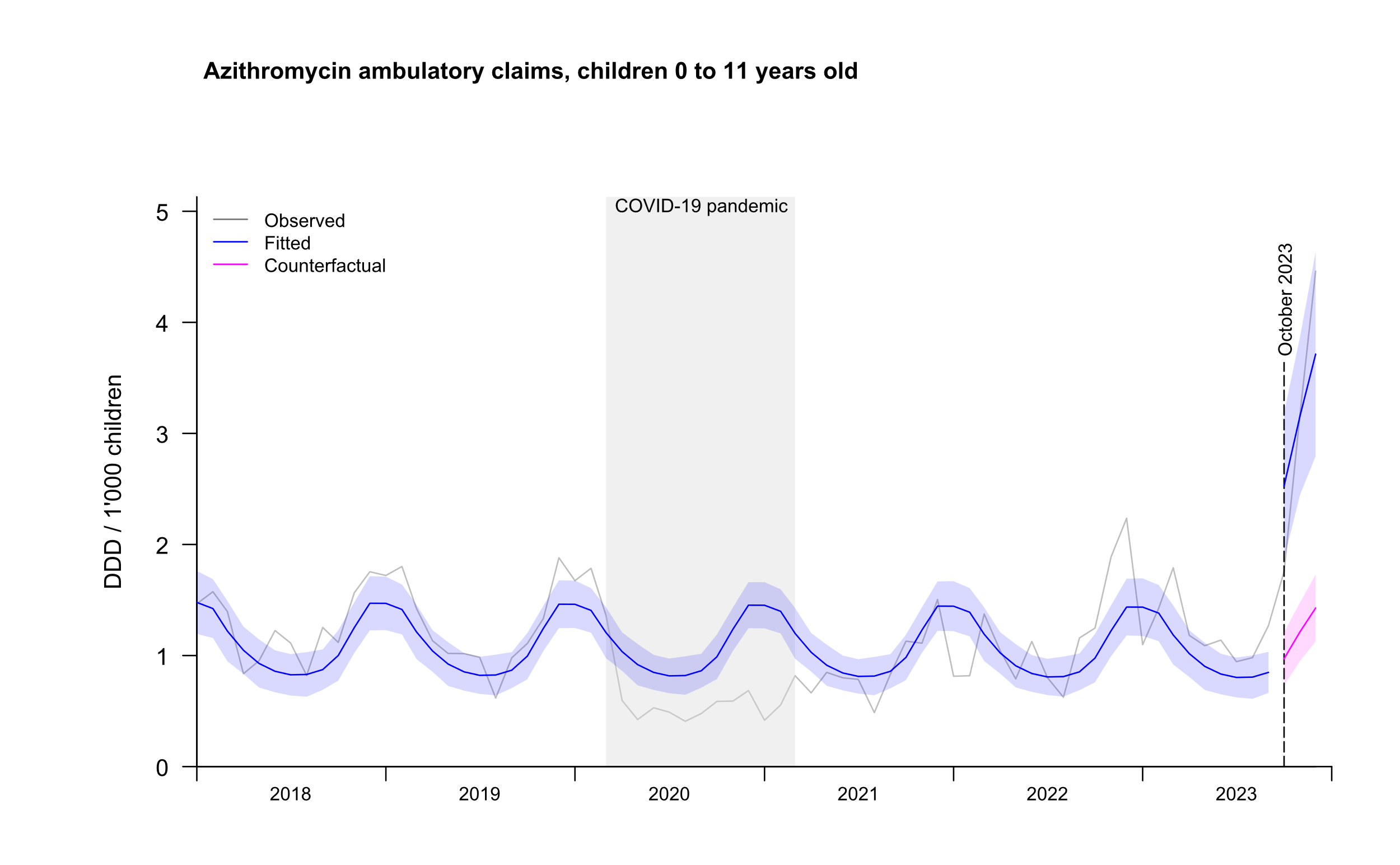


## Figure S7


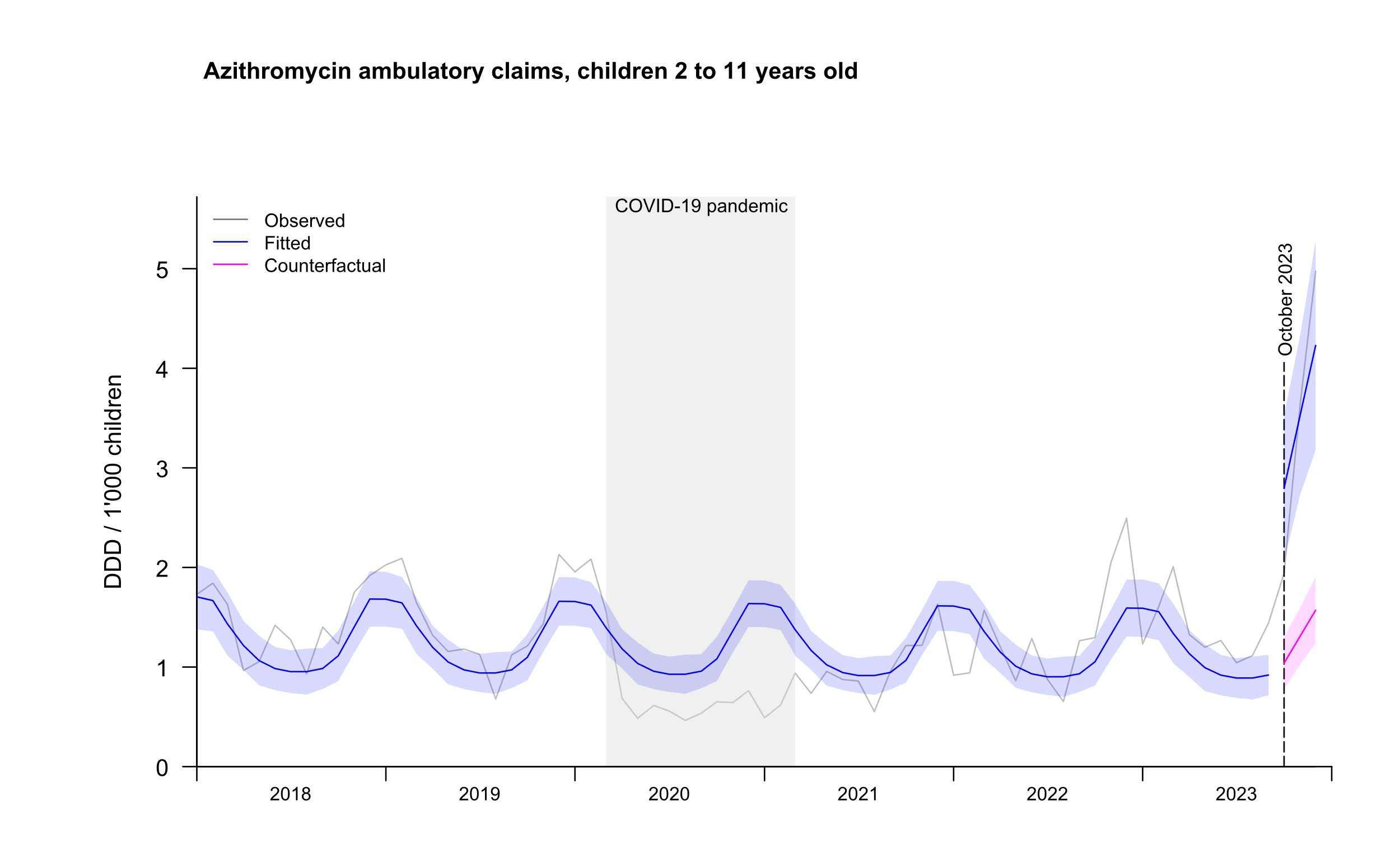


## Figure S8


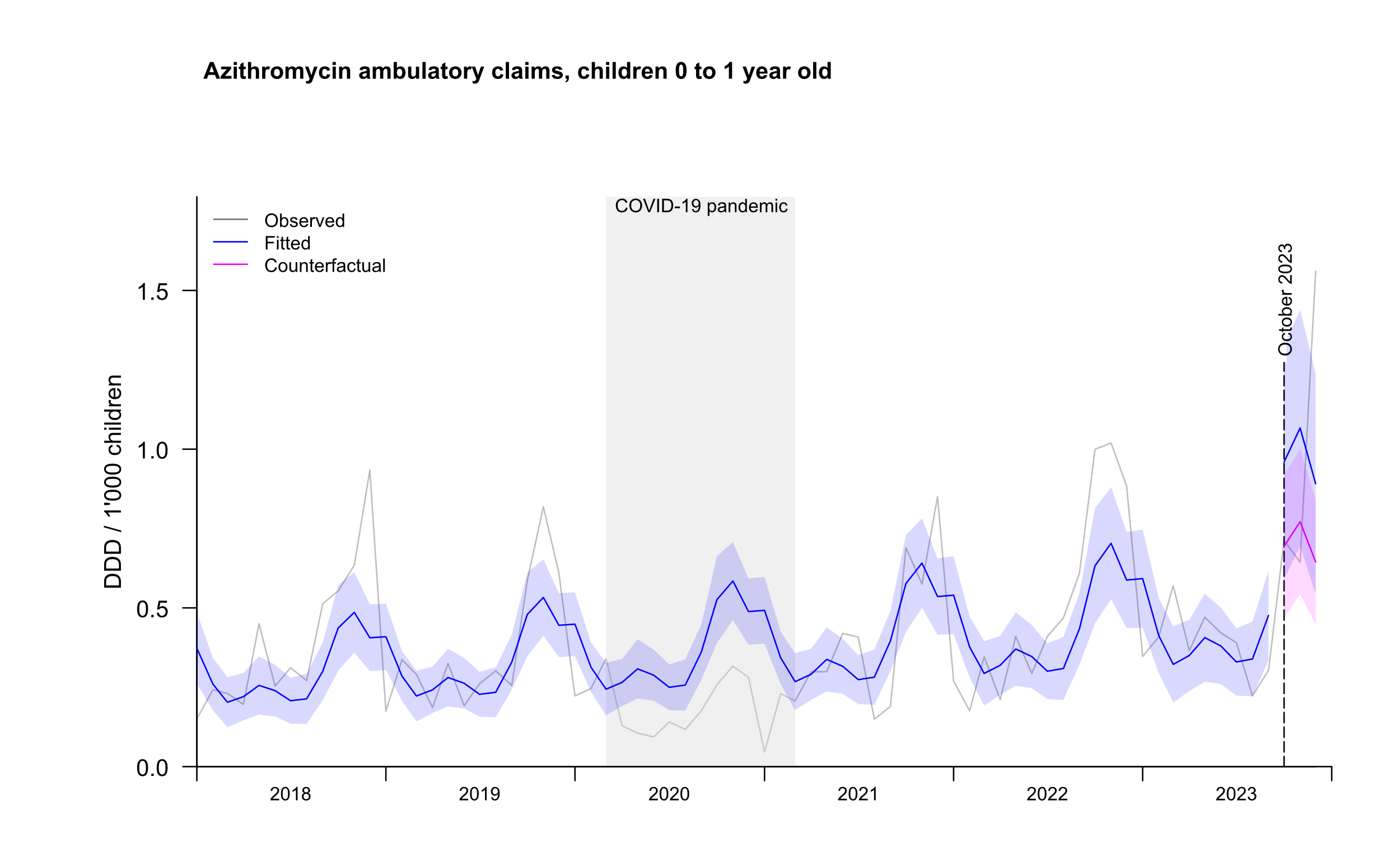


# Amoxicillin ambulatory consumption by age group (control outcome)

Monthly ambulatory Defined Daily Doses (DDDs) of amoxicillin per 1’000 children aged 0 to 11 years (Figure A1), 0 to 1 year (Figure A2) and 2 to 11 years (Figure A3).

The grey lines show the observed data. The blue line shows the fitted model based on observed data, with blue shaded area showing the 95% confidence intervals. The pink line shows the expected DDD based on data before October 2023, with pink shaded area showing the 95% confidence intervals.

## Figure S9


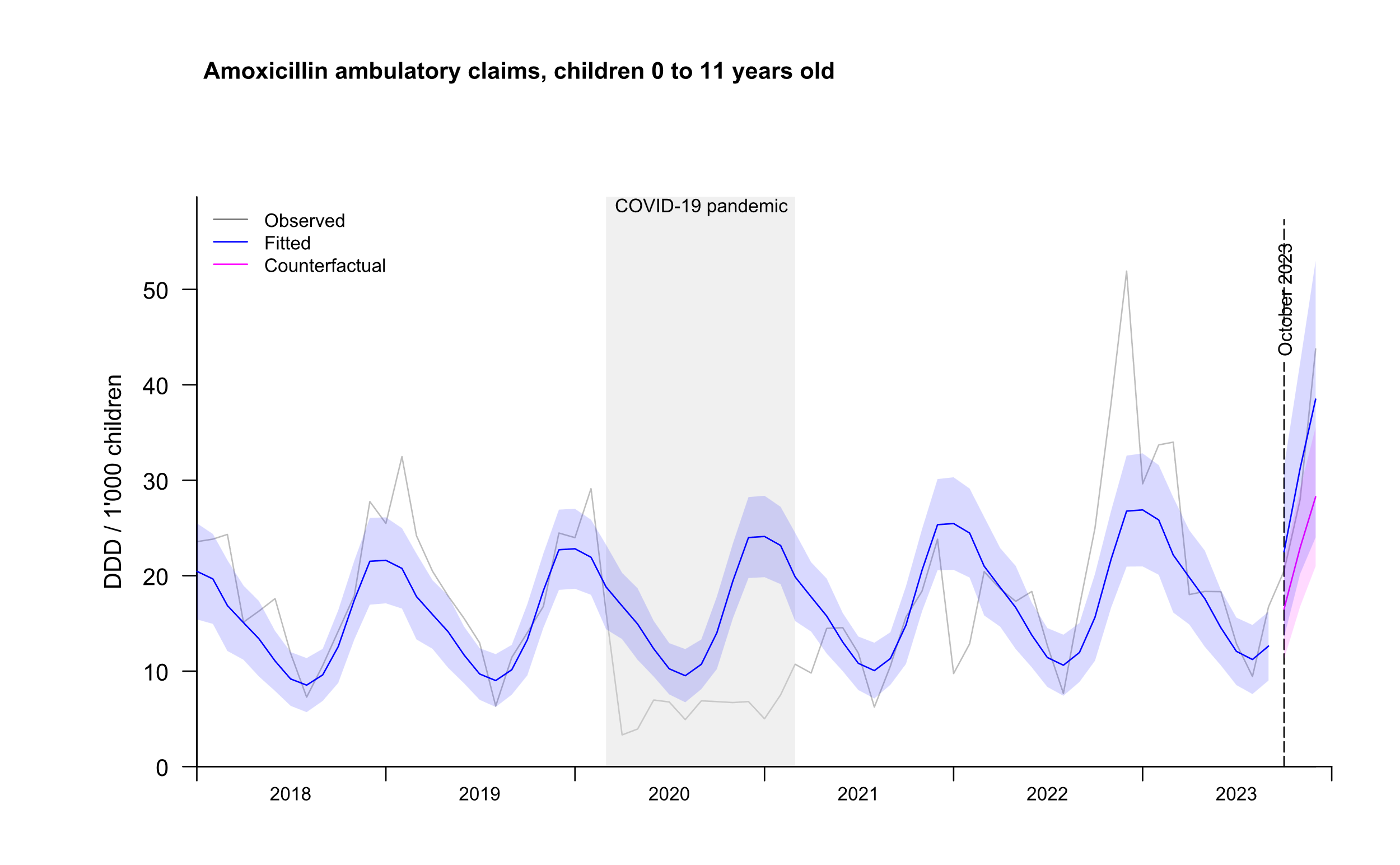


## Figure S10


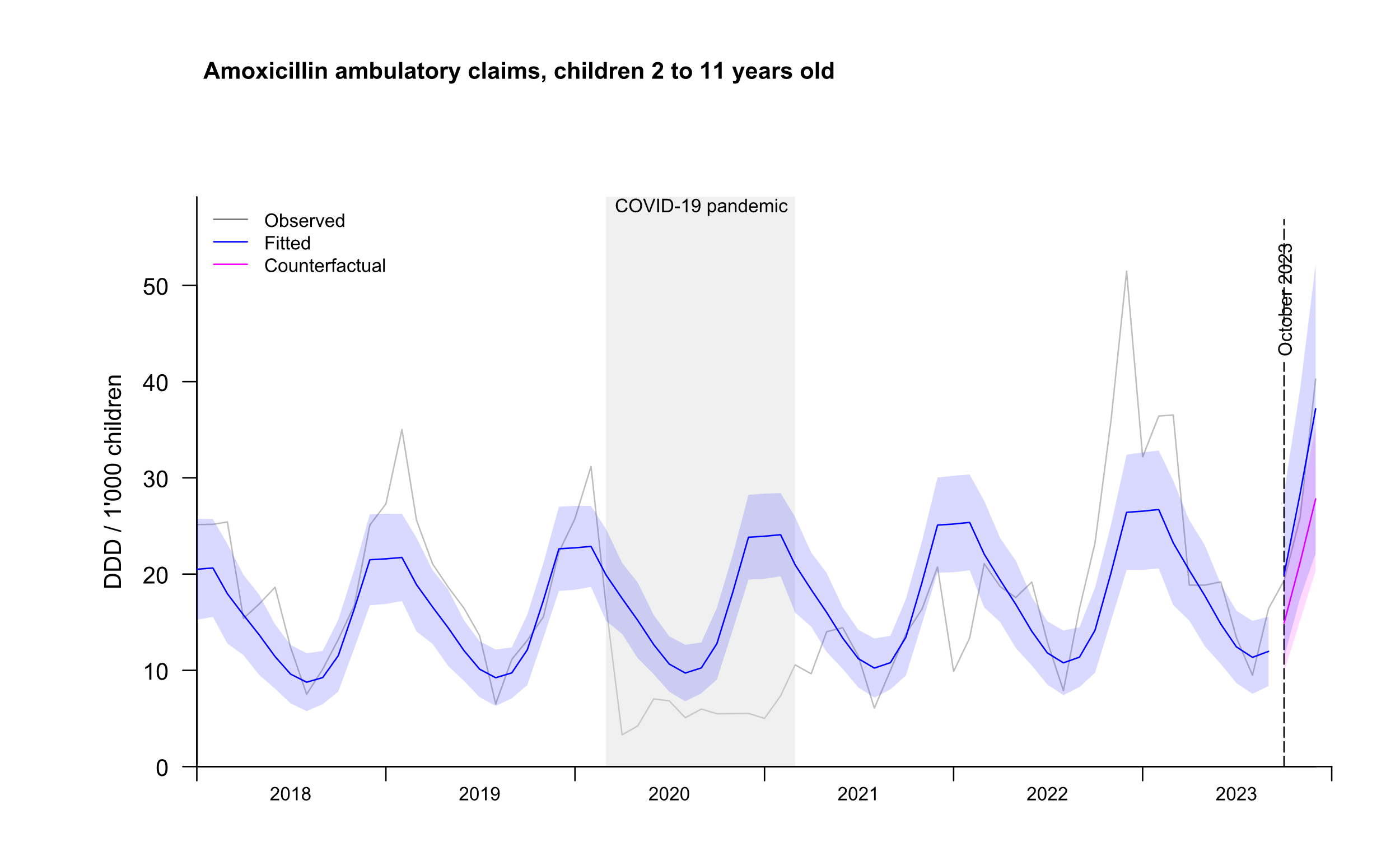


## Figure S11


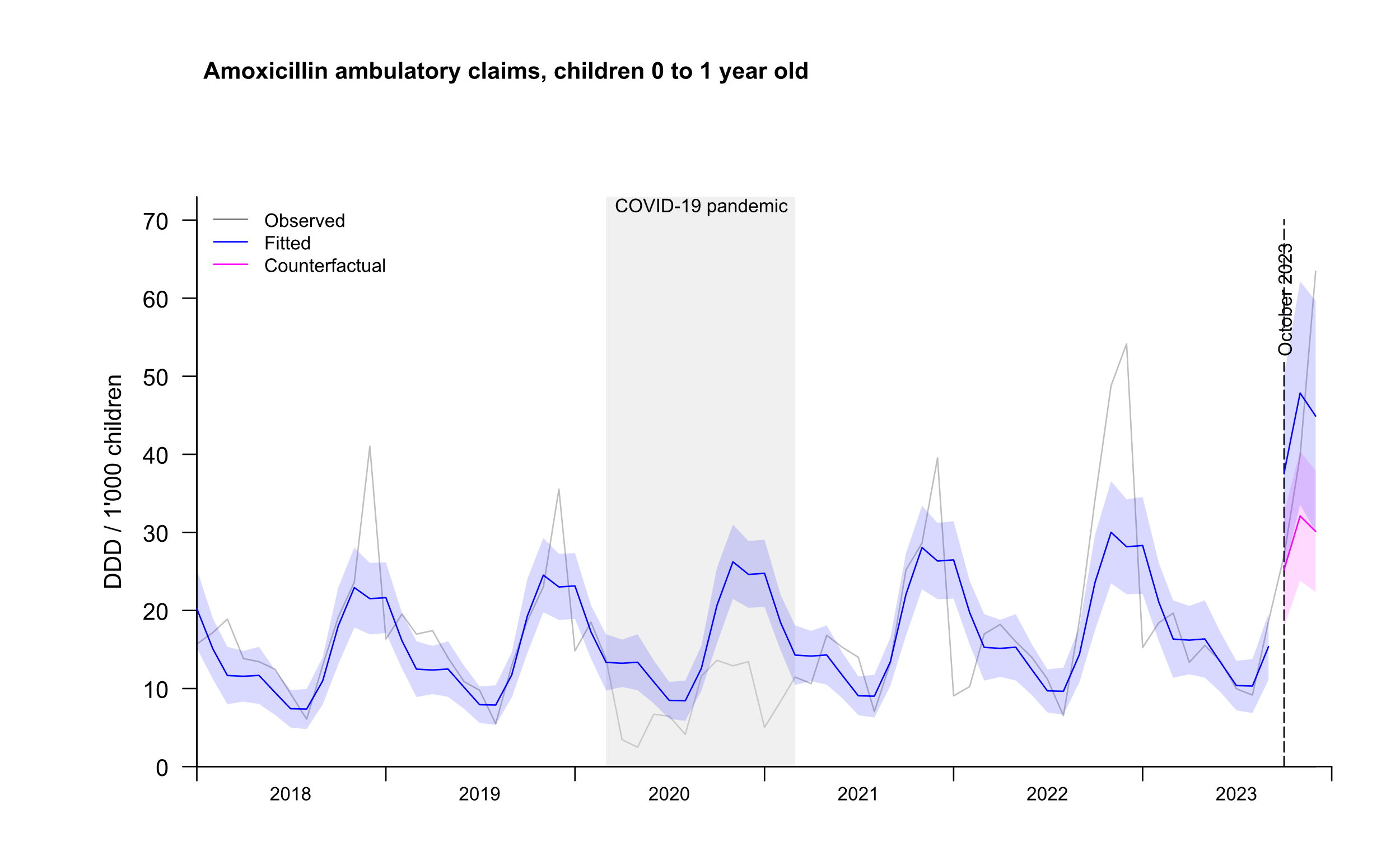

Supplement: dlaf123_Supplementary_Data [file dlaf123_supplementary_data.docx]
